# Rapid evolution of mammalian APLP1 as a synaptic adhesion molecule

**DOI:** 10.1038/s41598-021-90737-y

**Published:** 2021-05-28

**Authors:** Wataru Onodera, Toru Asahi, Naoya Sawamura

**Affiliations:** 1 Faculty of Science and Engineering, Waseda University, TWIns, 2-2 Wakamatsu, Shinjuku, Tokyo 162-8480 Japan; 2 Research Organization for Nano & Life Innovation, Waseda University, #03C309, TWIns, 2-2 Wakamatsu, Shinjuku, Tokyo 162-8480 Japan; 3 Green Computing Systems Research Organization, Waseda University, Shinjuku, Japan

**Keywords:** Molecular evolution, Coevolution

## Abstract

Amyloid precursor protein (APP) family members are involved in essential neuronal development including neurite outgrowth, neuronal migration and maturation of synapse and neuromuscular junction. Among the APP gene family members, amyloid precursor-like protein 1 (APLP1) is selectively expressed in neurons and has specialized functions during synaptogenesis. Although a potential role for APLP1 in neuronal evolution has been indicated, its precise evolutionary and functional contributions are unknown. This study shows the molecular evolution of the vertebrate APP family based on phylogenetic analysis, while contrasting the evolutionary differences within the APP family. Phylogenetic analysis showed 15 times higher substitution rate that is driven by positive selection at the stem branch of the mammalian APLP1, resulting in dissimilar protein sequences compared to APP/APLP2. Docking simulation identified one positively selected site in APLP1 that alters the heparin-binding site, which could affect its function, and dimerization rate. Furthermore, the evolutionary rate covariation between the mammalian APP family and synaptic adhesion molecules (SAMs) was confirmed, indicating that only APLP1 has evolved to gain synaptic adhesion property. Overall, our results suggest that the enhanced synaptogenesis property of APLP1 as one of the SAMs may have played a role in mammalian brain evolution.

## Introduction

Amyloid precursor protein (APP) family comprises of type-I single-pass transmembrane proteins and is composed of APP, amyloid precursor-like protein (APLP) 1, and APLP2. APP has been extensively studied in neurodegenerative diseases, especially Alzheimer’s disease, as its alternative cleavage generates the amyloid-β peptide; this peptide is found in the senile plaques of patients with Alzheimer’s disease^1^. All APP family members contain large ectodomains, each comprising the E1 and E2 domains, and a relatively smaller intracellular domain (Fig. 3a). The E1 domain is further subdivided into two subdomains: heparin-binding domain (HBD) and copper-binding domain (CuBD). These subdomains, bind to extracellular factors such as heparin, copper, or zinc ions, and secreted glycoproteins, including fibulin1^1^. Two heparin-binding sites are present in the HBD of E1 and E2; these sites are considered lost in APLP1 (Fig. 3a). The intracellular domain contains the YENPTY motif, which is conserved from *Drosophila* APP (APPL) to the vertebrate APP family, retaining several binding partners, including X11, FE65, and clathrin for endocytosis^1,2^. Kunitz-type protease inhibitor domain (KPI) is present between the E1 and E2 domains that is absent from APLP1.

Although the physiological functions of APP family, such as synaptic plasticity, synaptogenesis, neuronal protection, and maturation of the neuromuscular junction have been reported, a clear association of these functions with disease onset remains elusive^3–6^. Importantly, there exists redundancy as well as differentiation of APP, APLP1, and APLP2 functions. A complementary role was shown when contrasting brain development of triple knockout (KO) mice and single or double KO mice. In the triple KO mice, the cortical dysplasia characterized by focal ectopic neuroblasts had migrated through the basal lamina and pial membrane, whereas the single or double KO mice showed no abnormalities in cell organization during brain development in any of the mutants, thus, showing overlapping APP family functions^7,8^. However, distinct roles for APP family were suggested based on aged-single KO mice that retained different electrophysiological responses related to synaptic dysfunction: long term potentiation (LTP) was reduced in APP^−/−^ mice; spine density was reduced in APP^−/−^ and APLP1^−/−^ mice; and fiber volley/ excitatory post-synaptic potential (EPSP) slope was reduced in APLP1^−/−^ mice^3,9,10^. Furthermore, although double KO, APP^−/−^ APLP2^−/−^, and APLP1^−/−^ APLP2^−/−^ were both lethal owing to deficits in the neuromuscular junction, APP^−/−^ APLP1^−/−^ mice were viable, clearly showing unique roles for each family member^8^.

APLP1 specificity for central nervous system (CNS) is one of the differentiated functions of APP family members^11,12^. For example, APLP1 is expressed selectively in neurons, whereas APP and APLP2 are ubiquitously expressed^3,11^. More precisely, within the developing cortex where all APP family members are expressed, APLP1 is expressed exclusively in the cortical plate (CP), which later becomes layer II-VI in the mature brain^13^. An interacting partner of APLP1 at the presynaptic fraction, NB-2/contactin-5, has been identified^14^. The physiological function of APLP1 is also modified; compared with APP and APLP2, APLP1 has an augmented synaptogenic function. This function had been demonstrated through the co-culture of neurons and non-neuronal HEK293T cells overexpressing APP family members. Presynaptic formation was induced through contact with an axon. Among the APP family members, APLP1 exhibited the most efficient induction of synapses^3,4,15^. Based on this function, the APP family is classified as a synaptic adhesion molecule (SAM), fulfilling synaptic localization/function and cell adhesion property observed in proteins such as neuroligin and neurexin. Collectively, the unique expression and function of APLP1 in neurons may be considered a trait acquired after branching from the common ancestor of the APP family. However, there is uncertainty regarding the evolutionary process of APLP1 when acquiring the neuron-biased properties.

This study demonstrates the functional divergence of APLP1 from vertebrate APP/APLP2 using phylogenetic reconstruction and positive selection analysis. We showed that APLP1 evolves faster than APP/APLP2. Notably, significant evolution in APLP1 occurs at the stem of the mammalian APLP1, changing its protein properties, including a heparin-binding site for dimerization, distinct from that in the vertebrate APP family. In silico analysis of the heparin-binding site revealed a cavity solely in the mammalian APLP1-HBD, presumably sufficient for stable heparin-binding. Finally, a significant rise in evolutionary rate covariation was observed between mammalian APLP1 and SAMs, indicating the evolutionary direction of mammalian APLP1 as SAMs. The function of APLP1 as SAMs perhaps played a role in mammalian brain evolution.

## Results

### APLP1 distinct functions may have evolved from the stem branch of mammals

**Figure 1:**
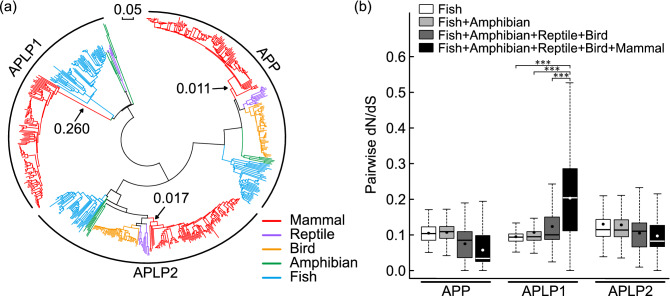
Evolutionary rate analysis for the vertebrate APP family. (**a**) The phylogenetic tree of the vertebrate APP family (nucleotide sequence) was constructed using the neighbor-joining method. Branch colors represent corresponding groups. Arrows point to the common ancestor of the mammalian branch, which shows longer branch length for APLP1 (APP = 0.011, APLP1 = 0.260, APLP2 = 0.017). (**b**) Pairwise dN/dS analysis of the represented groups in the vertebrate APP family. Dots indicate the mean of each dN/dS value. dN/dS was significantly larger for APLP1 when including mammalian clade while no such trend was observed for APP/APLP2 (***P-value < 0.01).

As APLP1 has specialized molecular functions and expression patterns after genetic duplication from the APP family, we hypothesized that it might have undergone accelerated evolution^16^. Based on the nucleotide sequences, a phylogenetic tree was constructed for 590 vertebrate APP family sequences (APP: 212, APLP1: 177, APLP2: 201) using neighbor-joining method^17^. Human APP family sequences were used as query sequences, and all the redundant sequences were cut after alignment (see Supplementary Table 1 online). As reported, APP family was detected in class of mammal, bird, reptile, amphibian, and fish, while APLP1 was not found in birds^2^. Interestingly, the stem branch of mammalian APLP1 exhibited about 15 times longer branch (APLP1 = 0.260, APP = 0.011, APLP2 = 0.017) compared to that of APP or APLP2, indicating rapid evolution of ancestral mammalian APLP1 (Fig. 1a). In addition, the branching order of mammal, bird, reptile, amphibian, and fish is irregular for APLP1, though APP and APLP2 branching were based on the species tree.

To evaluate the selective pressure on the stem of mammalian APLP1, the ratio of dN (substitution altering amino acid) and dS (substitution not altering amino acid or neutral substitution) was calculated^18,19^. First, we prepared four multiple sequence alignments (MSAs) of vertebrate APP family containing fish, fish + amphibian, fish + amphibian + reptile + bird, and fish + amphibian + reptile + bird + mammal, respectively. Then dN/dS between all the possible MSA pairs were compared statistically for each of the APP family members. As a result, unlike APP or APLP2, APLP1 showed a significant increase in dN/dS when including mammals in MSA (Fig. 1b). Furthermore, MSAs that are composed of only mammals were created for calculating intra-mammalian dN/dS. Intra-mammalian dN/dS of APLP1 did not exhibit the highest dN/dS value, suggesting APLP1 has accelerated substitution rate only at the stem of mammals (see Supplementary Fig. S1 online). Next, to examine branch-specific evolution at the stem of mammalian APLP1, a branch-site model based on maximum likelihood, dN/dS estimation was applied^19^. As a result, fitness to positive selection model (Model A) was significantly larger than its nested-null hypothesis (Model A null) at the stem branch of mammalian APLP1, supporting branch-specific positive selection, in contrast to APP or APLP2 (Table 1).

**Table 1:** Branch-site model applied to each stem branch of mammalian clade (*** p-value < 0.01; p-values are derived from likelihood ratio tests under test statistic 2Δℓ following χ^2^ distribution).

	Branch-site model	Ln L^a^	Model compared	LRT p-value	Sites under positive selection
APP	Model A	− 73,370.1	Model A vs Model A null	1	
	Model A null	− 73,370.1			
APLP1	Model A	− 70,196.9	Model A vs Model A null	< 0.001***	53S, 58G, 67R, 80R, 90R, 93E, 96R, 122G, 129A, 130H, 134Q, 142P, 155G, 171T, 172R, 175Q, 187I, 191S, 218P, 222A, 225D, 236R, 249F, 260P, 261P, 304I, 309G, 323R, 330R, 334M, 377A, 387Q, 446R, 450H, 521P, 525P, 531Q, 535S, 539E, 545E, 557R, 558G, 559F, 560P, 575G, 578V, 579S, 584S, 595S, 600S, 636R, 645R, 650R, 651P
	Model A null	− 70,219.3			
APLP2	Model A	− 85,255.6	Model A vs Model A null	0.526	
	Model A null	− 85,255.6			

^a^Log likelihood.

We also assessed the impact of genomic but not protein evolution on the rapid evolution of the stem of mammalian APLP1. This study estimated the GC-content (GC%) and the relative synonymous codon usage (RSCU), which can be under natural selection as it positively correlates with the expression of a gene for the efficiency of transcription or translation^20,21^. As a result, GC% of the vertebrate APP family varies from class to class (see Supplementary Fig. S2 online). GC content at third codon position (GC3%), which reflects GC-biased selection, was relatively high in some classes (APP-fish, APLP1-mammal, APLP1-reptile, APLP2-mammal, and APLP2-fish). However, high GC3% was not restricted only to APLP-mammal. Principle component analysis (PCA) was performed based on RSCU for the vertebrate APP family. APP, APLP1, and APLP2 were isolated to some extent, showing divergence on RSCU not only for APLP1, but also for the other APP family members (see Supplementary Fig. S3 online). Altogether, GC-content and RSCU vary among vertebrate APP families, supporting the notion that the protein sequence of APLP1 has extensively evolved at the stem of mammal.

### Mammalian APLP1 has a discrete protein sequence compared to other vertebrate APP families

**Figure 2:**
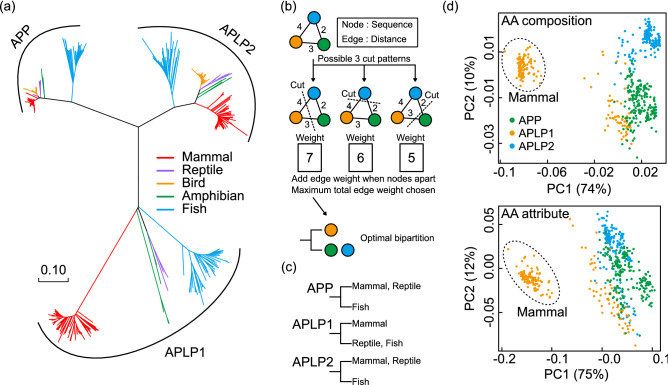
Difference in protein sequences of mammalian APLP1 among APP family. (**a**) The phylogenetic tree of the vertebrate APP family (protein sequence) was constructed using the neighbor-joining method. The mammalian stem for APLP1 shows a longer branch compared to APP/APLP2 consistent with nucleotide phylogenetic tree. (**b**) Overview of the max cut problem: the edges (distance) between partitioned nodes (sequence) are summed. The solution to the problem is to maximize the edges by searching for an optimal combination of bipartition. (**c**) Max cut problem was applied to the clustering protein sequence of APP family using digital annealer (Fujitsu, Japan). While mammalian APLP1 was clustered with the fish APLP1. (**d**) PCA applied to protein sequences of vertebrate APP family. 2 panels show the PCA of APP family described by amino acid (AA) composition and AA attribute, respectively. Descriptors for each sequence were calculated at ProtrWeb. Sequence in dotted circle represent mammalian APLP1, isolated from the other APP family.

To precisely differentiate the mammalian APLP1 protein sequence from the APP family, three distinguished clustering methods were applied to the same vertebrate APP family dataset. Figure 2a shows the phylogenetic tree of APP family protein sequences based on the neighbor-joining method, which clusters sequences from the closest pair branches^17^. It clearly showed distant mammalian APLP1 clade in contrast to mammalian APP and APLP2. This result is consistent with nucleotide sequence phylogenetic tree in Fig. 1a, suggesting nonsynonymous substitution substantially occurred at the stem branch of mammalian APLP1.

Subsequently, we conducted a pairwise sequence alignment (PSA) based clustering of the APP family sequences using the Max-cut algorithm to complement the above-mentioned phylogenetic analysis. In previous studies, it was observed that the interior branches close to the tree stem tend to reconstruct better when the evolutionary distance matrix is derived from the PSA, not from the MSA^22,23^. Additionally, from the same group, accurate sequence clusters were reported when using the graph-cut algorithm, as opposed to the neighbor-joining method^22^. Hence, Max-cut, a graph-cut algorithm, was applied to each of the PSA matrix of the APP family paralogs, which were comprised of 20–24 sequences each (Fig. 2b,c). The algorithm attempts to maximize the total distance between the two clusters (i.e. obtain two clusters that discriminates between a set of sequences). Thus, we were able to obtain results consistent with that of the neighbor-joining method in Fig. 2a. Amniotes (mammal-bird-reptile) and other vertebrates (amphibian-fish) were clustered with regards to APP and APLP2, showing a phylogenetically reasonable grouping. However, APLP1 exhibited clusters between mammals-fish and birds-reptiles-amphibians, suggesting a dissimilarity in the APLP1 sequences among the amniotes, as illustrated in Fig. 2a.

To determine if the protein sequences of mammalian APLP1 have specific properties distinct from other sequences, the sequences were clustered based on their amino acid composition grouped by attributes. Seven attributes (hydrophobicity, normalized Vander Waals volume, polarity, polarizability, charge, secondary structure, and solvent accessibility) as descriptors were calculated for each sequence^24,25^ and clustered using PCA. The top panel in Fig. 2d shows PCA of the vertebrate APP family written by simple amino acid composition. The lower panel in Fig. 2d shows the PCA of clustering sequences based on seven attributes as descriptors. As a result, the mammalian APLP1 was distinguished solely from other members of the vertebrate families, suggesting it has a unique amino acid sequence (Fig. 2d). Factor loadings in PCA revealed amino acids with intermediate polarity (P, A, T, G, S) and strand inducing amino acids (V, I, Y, C, W, F, T), which negatively and positively isolated mammalian APLP1 clusters (see Supplementary Table 2 online). Altogether, the mammalian protein APLP1 evolution might have been selected for a specific evolutionary axis.

### The heparin-binding domain of mammalian APLP1 undergoes a higher degree of positive selection

**Figure 3:**
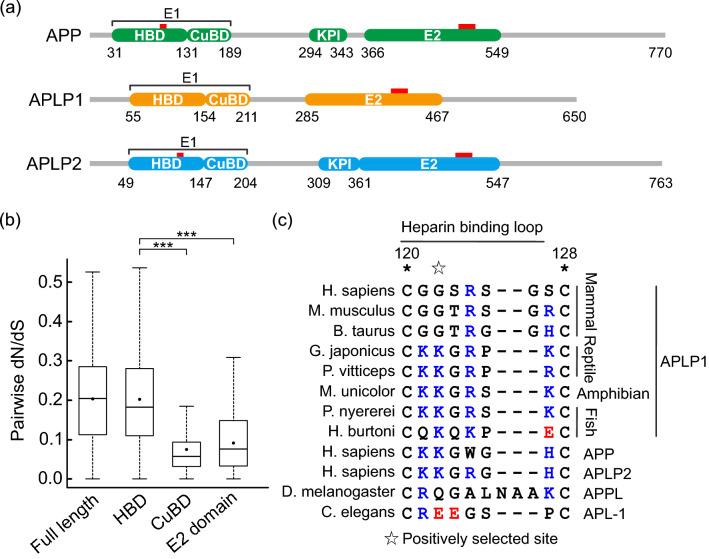
Positive selection of the heparin-binding domain of the mammalian APLP1. (**a**) The domain structure of the human APP family; heparin-binding domain (HBD); copper-binding domain (CuBD); Kunitz-type protease-inhibitor domain (KPI). Heparin binding sites are labeled with red line. (**b**) Pairwise dN/dS analysis of domains in the vertebrate APLP1. Dots show means of each dN/dS. HBD showed a significant increase in dN/dS. (***P-value < 0.01). (**c**) The positively selected sites for the stem branch of mammalian APLP1 detected by the branch-site model of dN/dS estimation. A site on the heparin-binding loop was selected, where the mammalian APLP1 has a glycine, while other sequences have positively charged residues (colored in blue).

Site-specific dN/dS is useful for identifying which function of mammalian APLP1 has evolved. First, to extract the domain that may have experienced functional evolution, pairwise dN/dS was estimated for each domain of the vertebrate APLP1. The comparison showed a significant increase in dN/dS in HBD (Fig. 3b). This domain is a subdivision of the E1 domain and is one of the binding sites for proteins, metal ions, and heparin, thus, regulating cis- or trans-dimerization of the APP family^15,26–28^. One of the positively selected sites (position 122 in human APLP1 sequence) in HBD is present in heparin-binding loop structures in APLP1 as detected by the branch-site model (Table 1, Fig. 3c).

It is speculated that the positively charged loop of APP/APLP2 regulates dimerization through electrostatic interactions with heparin, a small extracellular molecule^29,30^. The trans-dimer of the E1 domain from the APP family bridges pre-synapse and post-synapse. As such, it provides a site to assemble subsequent additional synaptic proteins (e.g., X11) for synapse maturation^31^. In contrast, mammalian APLP1 lacks the positively charged residues compared to other vertebrate APLP1 and it is considered to have lost the heparin-binding ability (Fig. 3c)^32,33^. In humans, four out of the six positions in APP and APLP2 HBD loop are positively charged residues, while in APLP1, only one out of the seven positions is a positively charged residue. Additionally, the mammalian APLP1 does not possess a heparin-binding consensus sequence (XBBXB: B is a positively charged amino acid, X is any amino acid), similar to the other APP family members^35^. Hence, we propose that positive selection leads to the loss of heparin-binding to the APLP1 loop. However, earlier studies showed that E1 domain heparin-affinity is directed only for APP, but not for APLP1; as such, there is no direct evidence suggesting the loss of heparin-binding at APLP1-E1 domain^30,33^. Furthermore, APLP1 may have a greater chance of contacting extracellular heparin than APP and APLP2, as it mainly localizes at the cell surface^3,27^. Docking simulations were used to identify whether APLP1-HBD has heparin-binding ability.

**Figure 4:**
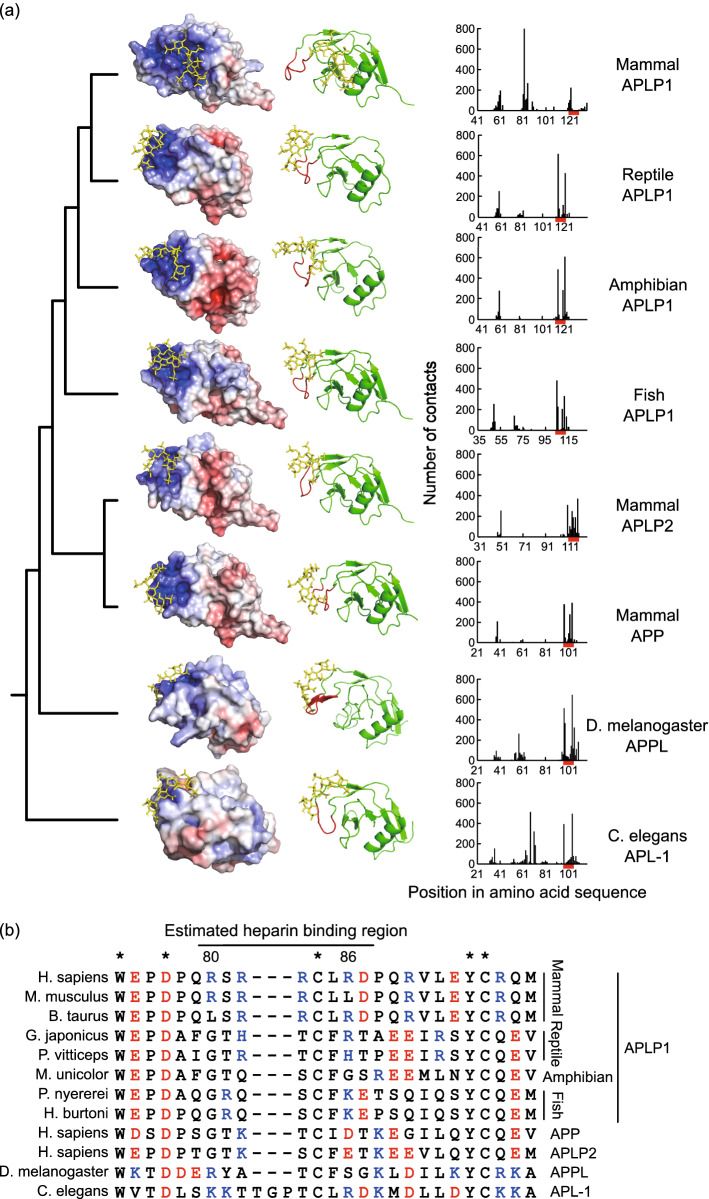
Functional simulation of the heparin-binding domain of the APP family. (**a**) The structure of HBDs with heparin along the phylogenetic tree of the APP family, including *C. elegans* and flies. The left column shows HBDs colored by surface charge (blue: positive charge, red; negative charge) and their binding to the heparin molecule (yellow). For all of the APP family, with the exception of the mammalian APLP1, heparin was bound to positively charged homologous protein surfaces by electrostatic interactions. The middle column presents the protein backbones. The red backbones indicate known heparin-binding sites; with the exception of mammalian APLP1, heparin binds around these sites. The right column shows a plot of the number of times heparin contacted a particular residue in an iteratively calculated docking simulation. A contact was defined as when heparin and the corresponding residue came within 4 Å of each other. The red bars indicate known heparin-binding sites. With the exception of the mammalian APLP1 and *C. elegans* APL-1, heparin most often contact with the known heparin-binding sites. The protein structures were visualized by PyMOL Molecular Graphics System, Version 2.3.2 Schrödinger, LLC. https://pymol.org. (**b**) MSA of the novel heparin-binding site revealed by docking simulation. In mammalian APLP1, a sequence similar to the binding consensus sequence of heparin appears (consensus XBBXB: B is a positively charged amino acid, X is any amino acid)^34^. Although positively charged amino acids were found in other species, they do not exist as a continuous motif.

Vertebrate APP family HBD structures were modeled based on homology modeling implemented in Phyre2 and subsequently docked with heparin using the ClusPro web server (template and input sequences are shown in Supplementary Table 3). Figure 4a (left column) shows heparin (colored in yellow) bound to the positively charged surface (blue) in vertebrate APP family. The middle column in Fig. 4a clearly illustrates the binding of heparin to the known heparin-binding loop (red), with the exception of mammalian APLP1. In contrast, mammalian APLP1, bound heparin at other positively charged surface. ClusPro quantified the results by counting the number of times heparin is bound to an arbitrary residue, where the contact is defined as distance < 4.0 Å. The quantification represented the emergence of novel heparin-binding residues (R80, R82, R83, and R86 in human sequence) conserved for mammalian APLP1 (Fig. 4a right column, Fig. 4b). Residues, R80, R82, and R83 of mammalian APLP1 are located in non-secondary structures, whereas R86 is present in a strand. Notably, R80 exhibited signs of significant positive selection, using the branch-site model (p-value = 0.002). Mammalian APLP1 has experienced loss of heparin-binding function at the loop and has subsequently acquired an alternative binding surface for heparin. Notably, APL-1 has a peak at a position similar to that of mammalian APLP1; while mammalian APLP1 has a consensus-like sequence for heparin-binding (XBBXBX), APL-1 has no such sequence. As this suggests low specificity of APL-1 and heparin-binding, the focus hereafter will be on mammalian APLP1^34^.

The heparin-binding site in the E2 domain was tested using a similar docking simulation procedure to identify if a mammalian APLP1 specific binding mode can be observed, as in HBD. This result of docking simulation detected key residues for heparin-binding found in the previous crystal structure of the E2 dimer-APLP1 (K422, H426, R429, H430, and H433)^35^. However, we did not detect extensive positional alterations of heparin for E2 docking as in mammalian APLP1-HBD (see Supplementary Fig. S4 and Supplementary Table 3 online). Comparing the homologous E2 sequence in the human APP family, the positively charged residues and the surrounding residues (position 407–430) were conserved. These observations suggest the conservation of the E2-heparin-binding sequence in the vertebrate APP family.

To clarify differences between the novel and known heparin-binding sites in mammalian APLP1, the focus shifted to the surface structure of the binding sites. Heparin interacts with proteins depending on the positively charged residues; this degree of interaction is affected by the nature on the grooves of the protein surface^36^. In particular, cavities and pockets of the protein structure contribute to the direction and strength of heparin binding^37^. The pocket-cavity search application (POCASA) web server was used to detect cavities in the HBD structure of mammalian APLP1. In POCASA, a sphere of an appropriate size is rolled onto a protein surface; the space where the sphere cannot not enter is defined as a cavity^38^.

**Figure 5:**
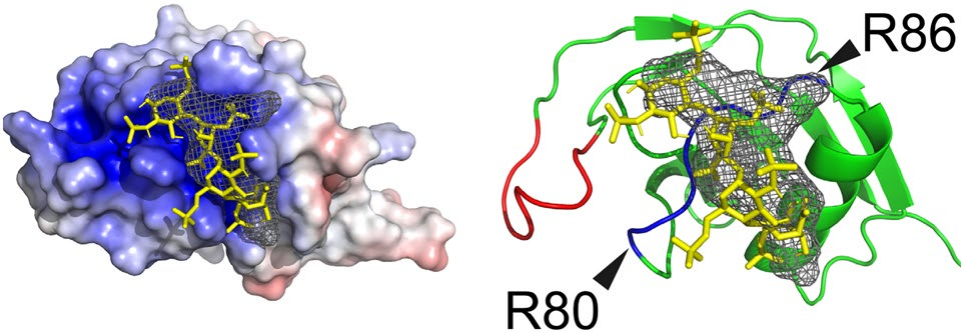
Cavity detection of HBD. The left panel shows HBDs colored by surface charge (blue: positive charge, red: negative charge) and their binding to heparin molecule (yellow). Detected cavity by POCASA is shown by the meshed structure. The cavity is positively charged and formed by novel heparin-binding site in blue (80–86) and adjacent α-helix. No cavity was detected for the known heparin-binding site in red (120–128). The protein structures were visualized by PyMOL Molecular Graphics System, Version 2.3.2 Schrödinger, LLC. https://pymol.org.

Using POCASA, a cavity was detected around the novel heparin-binding site (80–86) (Fig. 5: meshed region). This cavity was formed between the heparin-binding site (80–86) and the adjacent α-helix (87–97). The docking simulation results show that the heparin distribution corresponds to that of the cavity, suggesting that the direction of heparin chain binding may be specific. In addition, the bottom surface of the cavity was positively charged. These results suggest that the novel heparin-binding site provides the spatial and chemical conditions for heparin molecules to directionally bind at a deeper position on the protein surface. In contrast, there was no cavity detected around the known heparin-binding sites (120–128); this result may reflect heparin binding to occur superficially without any specific direction.

### Coevolutionary partners of mammalian APLP1 could be distinct from APP or APLP2

As shown above, the mammalian APLP1 evolutionary direction is indicated by the phylogenetic analysis. To further refine the mammalian APLP1 functional evolutionary direction, we conducted a coevolutionary analysis. The coevolutionary analysis is based on phylogenetic tree comparison, where trees tend to have similar topology when they experience similar evolutionary pressure for related functionality^39,40^. Here, the similarity of the phylogenetic trees is represented as a correlation coefficient and is defined as evolutionary rate covariation (ERC). ERC is derived from the comparison of each of the corresponding branch lengths after optimization based on species tree (details provided in “Methods”)^41^.

**Figure 6:**
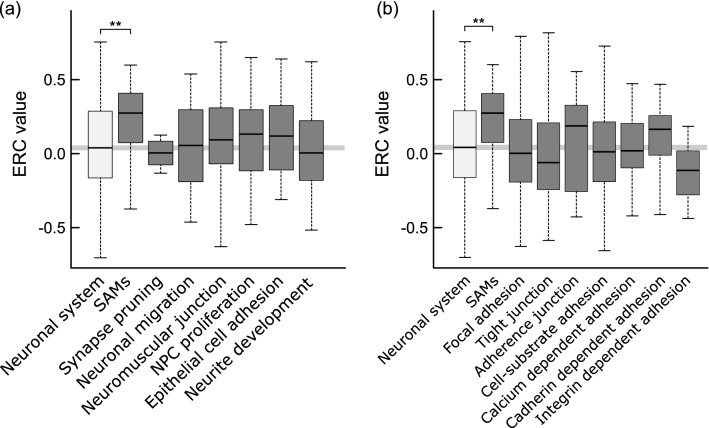
Comparison of coevolutionary analysis of APLP1, SAMs, and other neuronal-system related pathways. APLP1 has a statistically significant high ERC value with SAM proteins compared to that of neuronal system proteins. SAM: Synaptic adhesion molecule, NPC: Neural precursor protein. No such trend was detected for APP or APLP2 (Supplementary Table 4) (**P-value < 0.05 using one-tailed Dunnett’s test; regarding neuronal system as control group).

We focused specifically on SAM proteins, as APLP1 has enhanced cell–cell adhesion function at synapse compared to APP or APLP2, based on previous reports^3^. Moreover, our phylogenetic analysis and docking simulation support the evolution of mammalian APLP1 towards cell–cell adhesion (Figs. 3 and 4). ERC with mammalian APP family was calculated for reported 35 SAM and SAM-like proteins (e.g., neurexin, neuroligin, and calsyntenin3), and other neuronal-system related pathways including synapse pruning, neuronal migration, neuromuscular junction, neural precursor protein (NPC) proliferation, epithelial cell adhesion, and neurite development retrieved from the Reactome pathway or Gene Ontology^41–43^. In addition, we introduced a set of proteins including all neuronal system related proteins (R-HSA-112316: 329 genes) from the Reactome pathway. As it is the parental node that involves most of neuronal system related proteins, the ERC value between this gene set and APLP1 was used as basal ERC value. Subsequently, ERC values derived from each dataset were tested whether it shows statistically higher ERC values compared to the basal ERC value. As a result, the ERC value between SAM proteins and mammalian APLP1 was significantly higher compared to the basal ERC value. ERC values of APLP1 with the other pathway related proteins did not statistically exceed the basal ERC (Fig. 6a). This trend was absent for APP-SAM or APLP2-SAM ERCs (see Supplementary Table 4 online, APP-SAMs = − 0.009, APLP1-SAMs = 0.276, APLP2-SAMs = − 0.132). To confirm whether APLP1 coevolution depends on the specific type of cell adhesion or junction, its ERC values were compared with those of seven adhesion-related gene groups (Fig. 6b, Supplementary Table 5 online). None of the ERC values were significantly higher than the basal ERC values in any of the gene groups. As such, this ERC analysis revealed that the evolutionary profile of mammalian APLP1 is relatively close to SAM proteins.

## Discussion

Phylogenetic analysis, including phylogenetic tree reconstruction, positive selection analysis, and coevolution partner estimation, were applied to the vertebrate APP family. Vertebrate APP family tree had a branch that was 15 times longer at the stem branch of the mammalian APLP1 compared to corresponding branches of APP and APLP2. It was determined that the corresponding branch has undergone positive selection as demonstrated by the branch-site model of dN/dS calculation; and considered subsequently to result in unique protein sequences among vertebrate APP family, as revealed by PCA of amino acid composition grouped by attributes. To identify the functional divergence of mammalian APLP1, in silico heparin docking simulation was conducted by focusing on HBD that had the highest pairwise dN/dS value. As a result, the positively selected site in mammalian APLP1 HBD might lead to loss of heparin-binding; however, complemented in distinct binding site (positions 80–86). In addition, the novel site may have formed a cavity for heparin-binding. Finally, we used ERC values to refine the evolutionary direction of mammalian APLP1 as SAM.

The occurrence of the APLP1 branching event before APP/APLP2 branching was suggested previously by similar phylogenetic analysis^44^. Another phylogenetic analysis suggested a distant function of APLP2^2^. The differences in the results could be due to the analysis target, as they calculated a functional divergence of each paralog using all vertebrate sequences; this may mask the distinct evolution of the mammalian APLP1. Our analysis shows evolution acceleration at the stem of the mammalian APLP1, driven presumably by positive selection through the separate analysis of each vertebrate class. PCA results (Fig. 2d) show that the evolution of mammalian APLP1 resulted in protein sequences with specific protein properties, different from not only mammalian APP/APLP2, but also non-mammalian APLP1. The directional evolution of mammalian APLP1 could perhaps result in specialization towards mammalian neurons or brain; however, this does not apply for tissue-specific expression, since the APLP1 expression location has not changed from zebrafish (expression data for zebrafish is from https://zfin.org/ZDB-FIG-050630-2590). It is worth comparing the mammalian APLP1 with nonmammalian APLP1 on the basis of cell biology experiments, including tests for cell–cell adhesion, dimerization ratio, rate of endocytosis, and efficiency of synaptogenesis, the reported functional differences of APLP1 compared to mammalian APP or APLP2^3,45^.

Branch-site models implemented in the phylogenetic analysis by maximum likelihood (PAML) enabled us to further analyze the specific site under positive selection at the stem branch of mammalian APLP1 (Table 1, Figs. 3, 4). Substitutions at the heparin-binding loop in HBD in particular caused loss of heparin-binding function in the mammalian APLP1 lineage, which was complemented with another unique positively charged site at 80–86 position (Fig. 4). Interestingly, the site possesses a heparin-binding consensus-like sequence (XBBXB)^34^. Further investigation of the surface of the HBD enabled the discrimination of the mammalian APLP1 from the other APP family. A positively charged narrow cavity was detected that may serve as a tight heparin-binding site (Fig. 5). Notably, the residues constituting the cavity have low b-factor, indicating the cavity is inflexible^30^. The rigidity of the cavity may help stabilizing the heparin as such cavities are capable of stable hydrogen bonding with heparin^37^. In contrast, the binding to the known flexible heparin-binding loop may be promoted mainly by electrostatic interaction as it is disfavored by stable hydrogen bonds. Furthermore, such cavities tend to possess tightly bound conserved water molecules when unbound to ligands^46^. The desolvation upon heparin-binding contributes to a binding free energy and stabilize the complex apart from the electrostatic interaction^47,48^. Altogether, the ability of mammalian APLP1 to trap heparin through the acquisition of a novel heparin-binding site may contribute to the increased dimerization efficiency of APLP1, forming stable HBD-heparin complexes.

The KPI domain is a domain within the conserved APP family that is absent only in vertebrate APLP1. This domain is missing in some transcript variants, such as APP695. Although the KPI domain is not a direct binding surface for dimerization, it may promote dimerization through coordination of the ectodomain structure^49^. Therefore, it is unlikely that the absence of the KPI domain may be attributable to the increase in the efficiency of the APLP1 dimer formation. It is also not expected to be relevant to mammalian APLP1-specific evolution, as the entire APLP1 vertebrate lineage lacks the KPI domain.

The juxtamembrane/transmembrane domain (JM/TM) is a disordered region containing site recognized by secretases. Three GxxxG motifs are present in the JM/TM of APP; these are considered to promote APP cis-dimerization in the transmembrane region and reduce amyloid-β production. There is still an ongoing debate as to whether the C-terminal fragments of APP directly promote dimerization by GxxxG motifs; however, mutation of the GxxxG motif appears to have no effect on the dimerization of full-length APP^28,49–51^. As this motif is present in APLP1 (590-GAGGG-594), it may also contribute to the formation of the cis-dimer in APLP1. Interestingly, the GxxxG motif is specific to mammalian APLP1, and non-mammalian APLP1 appears to possess a similar motif, AxxxA (AVAIA). This motif also serves as a dimerization interface; however, it is less efficient in terms of the dimerization rate compared to GxxxG^52^. As cis-oligomers have been reported to bundle together to form trans-oligomers for APLP1^45^, the evolutionary change in cis-dimerization efficiency also needs to be considered. We did not detect any positive selection sites within this dimerization motif. However, further investigation is required to determine whether the evolution of the JM/TM domain contributes to the dimerization efficiency of APLP1. This is because a dimerization interface other than the GxxxG motif may participate within the alpha-helix of the transmembrane^49^.

The positive selection test revealed 16 positive selection sites in the JM/TM domains. Of these 16 sites, only three (S535, E539, and S594) had mammalian-specific amino acids, while the remainder had amino acids that differed between vertebrate classes and species. These variable sites may have been detected as false positives because disordered juxtamembrane domains may have evolutionary constraints related to the conservation of structure relaxed compared to other domains. Interestingly, among the three mammalian-specific positively selected sites, S594 is one of the residues recognized by γ-secretase^53^. The surrounding recognition sequences were also dissimilar when compared with mammals (593-GGSLI-597) and non-mammals (IAMVM). These evolutionary mutations require further investigation, as there is a strong association between the processing of the APP family and dimerization/oligomerization^54,55^.

Based on in vitro findings that APLP1 exhibits SAM properties, we evaluated whether the evolutionary profiles are also directed towards SAM, using coevolutionary information of known SAM proteins^3^. As a result, the phylogenetic tree of the mammalian APLP1 showed a significant correlation with other SAM proteins, suggesting global evolutionary pressure on mammalian APLP1 to develop into SAM specific sequence. Interestingly, the other neural or adhesion pathways did not show high ERC value, indicating APLP1’s particular evolution towards SAM proteins (Fig. 6). For APP and APLP2, coevolutionary signs between SAMs and the other neural or adhesion pathways were not able to be detected (see Supplementary Tables 4 and 5 online). As ubiquitously expressed proteins, such as APP and APLP2, tend to be evolutionarily constrained, they may not coevolve with a certain pathway. ERC analysis tends to detect not only binding partners, but also proteins in same pathway or similar function, requiring further experiments to determine how biologically related the coevolving proteins are for APLP1^39,40^. Notably, in the mouse brain, each member of the APP family has a unique set of binding partners^56^. This raises the possibility of acquisition of APLP1’s specific binding partners at stem of mammal, that may adjust evolutionary rate of APLP1 towards SAM proteins. Moreover, human APLP1 shows stronger localization at the cell surface and CNS compared to APP/APLP2, having considerable chance of interaction with binding partners which the other family members rarely coexpress^3,12^. In addition, the difference between transcriptional activities among APP family may be interesting to investigate as the APP tunes neurotransmission through transcriptional activity that is dependent on the cleaved intracellular domain^57,58^. Further investigations are needed to identify to what evolutionary demand mammalian APLP1 had responded. It is interesting to see how the accelerated evolution of APLP1 led the mammalian brain to achieve a unique six-layered cortex and expand the neocortex region among amniotes^59^.

## Methods

### Sequence data collection

Nucleotide sequences of 682 vertebrate APP family sequences were collected using megablast or BLASTn in May of 2018^60^. Human APP family was set as query sequences (APP: NM_000484, APLP1: NM_001024807, APLP2: NM_001642). Clustal W implemented in the molecular evolutionary genetics analysis (MEGA7) was used for generating protein MSA files using default parameters^61,62^. After manually removing overlapping species and non-aligned/partial sequences, a total of 590 sequences (APP: 212, APLP1: 177, APLP2: 201) remained for further analysis.

A 1:1 orthologous sequence of human APLP1 and APLP2 was chosen using the same procedure as described earlier, verified through the orthologous matrix (OMA) browser for a list of gene-level orthologues^63,64^. In contrast, fish APP linage seemed to independently experience duplication events resulting in duplicates named APPa and APPb^2^. As duplication may accelerate the heterogeneity of dN/dS value^65^, we compared dN/dS of APPa/b and concluded that their insignificant dN/dS values would not affect the results of the dN/dS analysis. The APPa/b sequences were collected as mentioned above using zebrafish APPa/b (APPa: NM_131564, APPb: NM_152886) as a query (total of 29 species each).

### Phylogenetic tree reconstruction

Phylogenetic trees were constructed using the neighbor-joining method implemented in MEGA7^17,45^. The evolutionary distance of the nucleotide sequence was calculated based on the maximum composite likelihood method^66^. Dayhoff matrix was used to calculate the evolutionary distance of the amino acid sequence^67^. Sites with gaps were not included in the reconstruction. To confirm the assurance of branching events, bootstrap value (resampling = 100 times) was inferred, and branches retaining bootstrap value > 50 are considered in the analysis^68^.

### Positive selection estimation using dN/dS calculation

A pairwise dN/dS calculation was performed in MEGA7 by Li-Wu-Luo method with 100 bootstrap replications for each APP family divided into either clade level (fish, fish + amphibian, fish + amphibian + reptile + bird, and fish + amphibian + reptile + bird + mammal) or domain level (HBD, CuBD, E2 domain)^69^. Sites with gaps were excluded from the calculation. Bootstrap resampling was conducted, as shown above.

Maximum likelihood dN/dS calculation under branch-site model (Model A vs. Model A null) implemented in PAML was performed as described earlier, using EasyCodeML that contains GUI-based format conversion and CodeML in one platform^70,71^. Here, MSA and phylogenetic tree data were prepared separately for each vertebrate APP family using a similar procedure as described above with three inputs. This method differs from pairwise dN/dS calculation due to its direct estimation of positive selection on a branch of interest (foreground branch) with statistical testing.

### Clustering method using Digital Annealer

Protein sequences of the APP family paralogs were clustered using the Fujitsu’s Digital Annealer (DA), a hardware specialized for solving optimization problems^72^. Briefly, given a sequence distance matrix and cost function, DA searches for optimal bipartition of the dataset to maximize the distance between the two clusters. The distance matrix (termed the similarity graph) was first described by the evolutionary distance using PSA. PSA and evolutionary distance (here, Poisson-corrected distance) calculation was performed using the Data Analysis in Molecular Biology and Evolution (DAMBE) software^23^. Thereafter, we assigned each sequence into an appropriate cluster and maximized the total distance between the two clusters; cost function was used to describe the total distance. As DA accepts binomial values for the variables, the total distance between two clusters containing n number of sequences was calculated as follows:

$$total \; distance= \sum_{i=1}^{n}\sum_{j>i}{D}_{ij}{({x}_{i}-{x}_{j})}^{2}$$

where ${x}_{i} \in \{\mathrm{0,1}\}$ is the $i$ th sequence of a variable and ${D}_{ij}$ is the evolutionary distance between sequences *i* and *j*. This formula only adds distance when two arbitrary sequences were assigned to different clusters. The bipartition found here was called the Max-cut. Algorithms of the calculation in DA are described in Aramon et al. 2019 (for implementation, refer to the DA webservice: https://www.fujitsu.com/global/services/business-services/digital-annealer/index.html). In this study we used a total of 20–24 sequences that were randomly selected from the sequences of the APP family in order to use equal numbers of sequences from each class.

### Principle component analysis (PCA) and dataset preparation

PCA was conducted for the vertebrate APP family sequence based on RSCU, amino acid composition, and amino acid composition grouped by attributes. RSCU and GC-content were calculated in MEGA7^17,45^. Seven attributes, hydrophobicity, normalized Vander Waals volume, polarity, polarizability, charge, secondary structure, and solvent accessibility and their composition for each sequence, were calculated using the ProtrWeb server^73^. Then, PCA using prcomp function in stats supplied as a default package in R was applied for the dataset. The results for ProtrWeb and PCA, including factor loadings, are shown in the Supplementary Table 2.

### Tertiary structure modeling, docking simulation and cavity detection of HBD

Tertiary structures of HBD of human APP family (APP: NM_000484, APLP1: NM_001024807, APLP2: NM_001642, reptile APLP1: XM_030212233.1, amphibian APLP1: XM_026702387, and fish APLP1: XM_012862627) were modeled based on homology modeling as implemented in Phyre2 server^74^. Briefly, each HBD of the APP family was truncated manually and subsequently submitted to the webserver. Alanine substitution model for human APLP1 was manually created by changing to R80A, R82A, R83A, and R86A. The default mode was used for tertiary structure prediction. Protein models with the confidence of 100% and percent identity > 40% were chosen for further prediction, which are preferred index values for reliable models. The template crystal structure for homology modeling was HBD of APP (PDB: chain A of 1MWP). Detailed inputs and output scores for modeling are presented in supplementary Table 3.

Subsequently, the predicted models were transferred to the heparin docking simulation implemented in ClusPro 2.0 server^75^. Briefly, the ligand (heparin) first determines 70,000 rotation poses and parallelly translates around fixed HBD protein structure by 1.0 Å 3D grid. Then, for each grid point, the pose with the lowest interaction energy (scored by van der Waals attractive, van der Waals repulsive, and electrostatic energy) is selected. Finally, among the selected poses, neighboring poses defined as RMSD within 9 Å are clustered to determine the most preferred binding mode chosen by its clustering size. Default parameters were used for heparin docking simulation. The surface electrostatics of protein models were calculated using the PDB2PQR server using a pH of 7.4^76^.

Cavity detection in the HBD of human APLP1 was conducted using the POCASA 1.1 webserver^38^. All parameters were set to default, with the exception of the probe radius which was adjusted to 4 Å. The visualization, including models, surface electrostatics and cavities was conducted in PyMOL Molecular Graphics System, Version 2.3.2 Schrödinger, LLC. https://pymol.org.

### Coevolutionary analysis using evolutionary rate covariation and coexpression

The coevolutionary analysis was undertaken using evolutionary rate covariation (ERC) value. ERC value is the degree of similarity in a pair of proteins based on phylogenetic tree similarities, here predicted in webserver^40^. The dataset is generated from all available mammalian protein sequences in the UCSC server. Briefly, the correlation coefficient derived from covariation of evolutionary distance between corresponding branches of pair proteins is calculated. Notably, this server normalizes the evolutionary distance by species tree before the calculation of the correlation coefficient.

### Statistical analysis

The mean dN/dS values for each group of sequences (either grouped by species: fish, fish + amphibian, fish + amphibian + reptile + bird, and fish + amphibian + reptile + bird + mammal or domain level: HBD, CuBD, E2 domain) were compared by multiple comparison using one-way ANOVA with posthoc two-tailed Tukey HSD Test (P-value: * < 0.10, ** < 0.05, *** < 0.01).

The fitness of model A and model A null were compared by likelihood ratio test (LRT) assuming test statistic 2Δℓ following χ^2^ distribution: positive selection model or Model A (allowing dN/dS or ω > 1 for foreground branches) and purifying selection model or Model A null (not allowing dN/dS or ω = 1 for foreground branches). Subsequently, Bayes Empirical Bayes (BEB) analysis were applied for sites in foreground branch with ω > 1 to test whether its value significantly have ω > 1 (positive selection) judged by posterior probability (Posterior probability: * > 0.90, ** > 0.95, *** > 0.99).

The mean ERC values were compared with the neuronal system’s ERC value using one-tailed Dunnett’s test (P-value: * < 0.10, ** < 0.05, *** < 0.01).

## Supplementary Information

Supplementary Information 1.

Supplementary Information 2.

## Data Availability

The datasets generated during and/or analyzed during the current study are available from the corresponding author on reasonable request.
