# Supplementary material for: Rapid evolution of mammalian APLP1 as a synaptic adhesion molecule
Source: Sci Rep. 2021 May 28;11:11305. doi: 10.1038/s41598-021-90737-y (PMC8163877; doi:10.1038/s41598-021-90737-y)

**Supplementary Materials for**

**Rapid evolution of mammalian APLP1 as a synaptic adhesion molecule**

Wataru Onodera, Toru Asahi, and Naoya Sawamura

Correspondence to: naoya.sawamura@gmail.com, naoya@aoni.waseda.jp

**Supplementary Figs. S1 to S4**

**Supplementary Fig. S1.**

Pairwise dN/dS for mammalian clade of APP family, represented here as intra-mammal pairwise dN/dS.


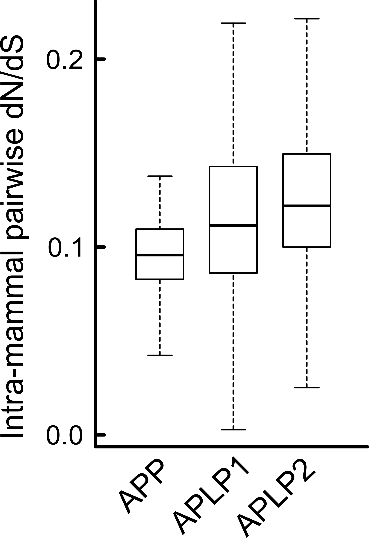


**Supplementary Fig. S2.**

GC% and GC3% was calculated for vertebrate APP family. Red dotted line represents GC% of 70%. Wide range of GC3% (37-86%) was observed reflecting diversity for GC content. Generally, each GC% are grouped by class-level order (e.g. APLP1-mammal).

**
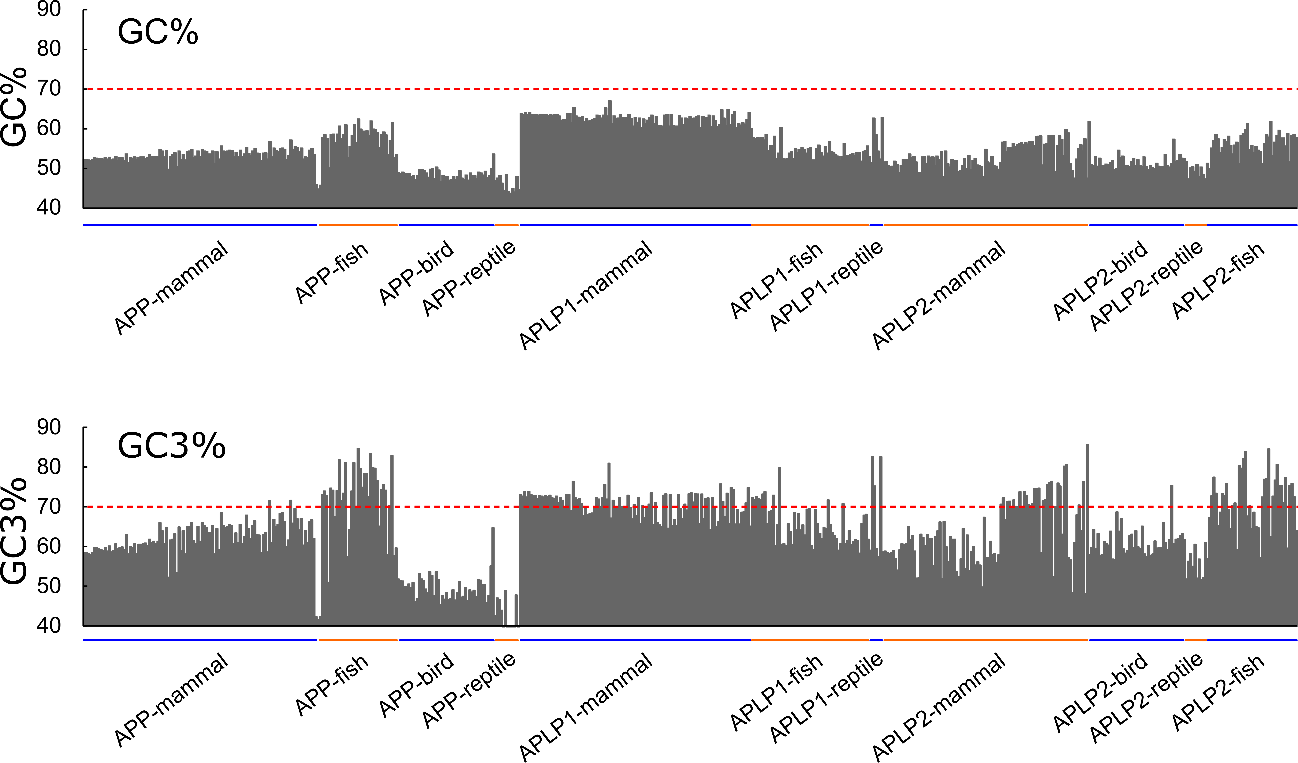
**

**Supplementary Fig. S3.**

PCA of Relative synonymous codon usage (RSCU) for APP family was plotted. Clear isolation of APLP1-mammal was not observed as in Fig 2 (d), indicating that RSCU is relatively conserved among APP family proteins.


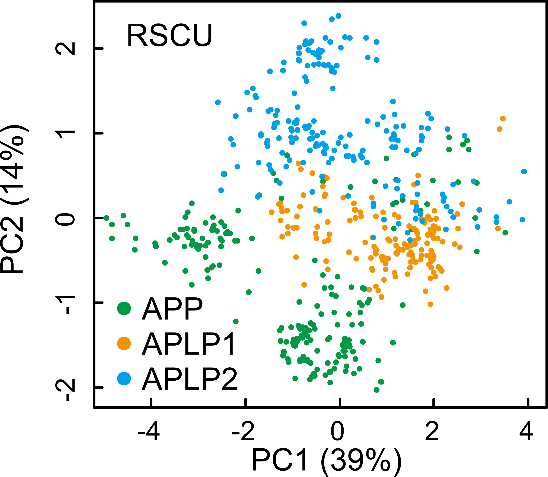


**Supplementary Fig. S4.**

Functional simulation of E2 domain of APP family. (a) Docking simulation of HBD and heparin. The surface charge of the HBD are exhibited with bound heparin (orange). The protein structures were visualized by PyMOL Molecular Graphics System, Version 2.3.2 Schrödinger, LLC. https://pymol.org. (b) Quantification of heparin and residue contacts (defined as counts of heparin and residue atoms < 4 Å). Heparin was bound at similar position of aligned APP family. (c) MSA of heparin bound positions.


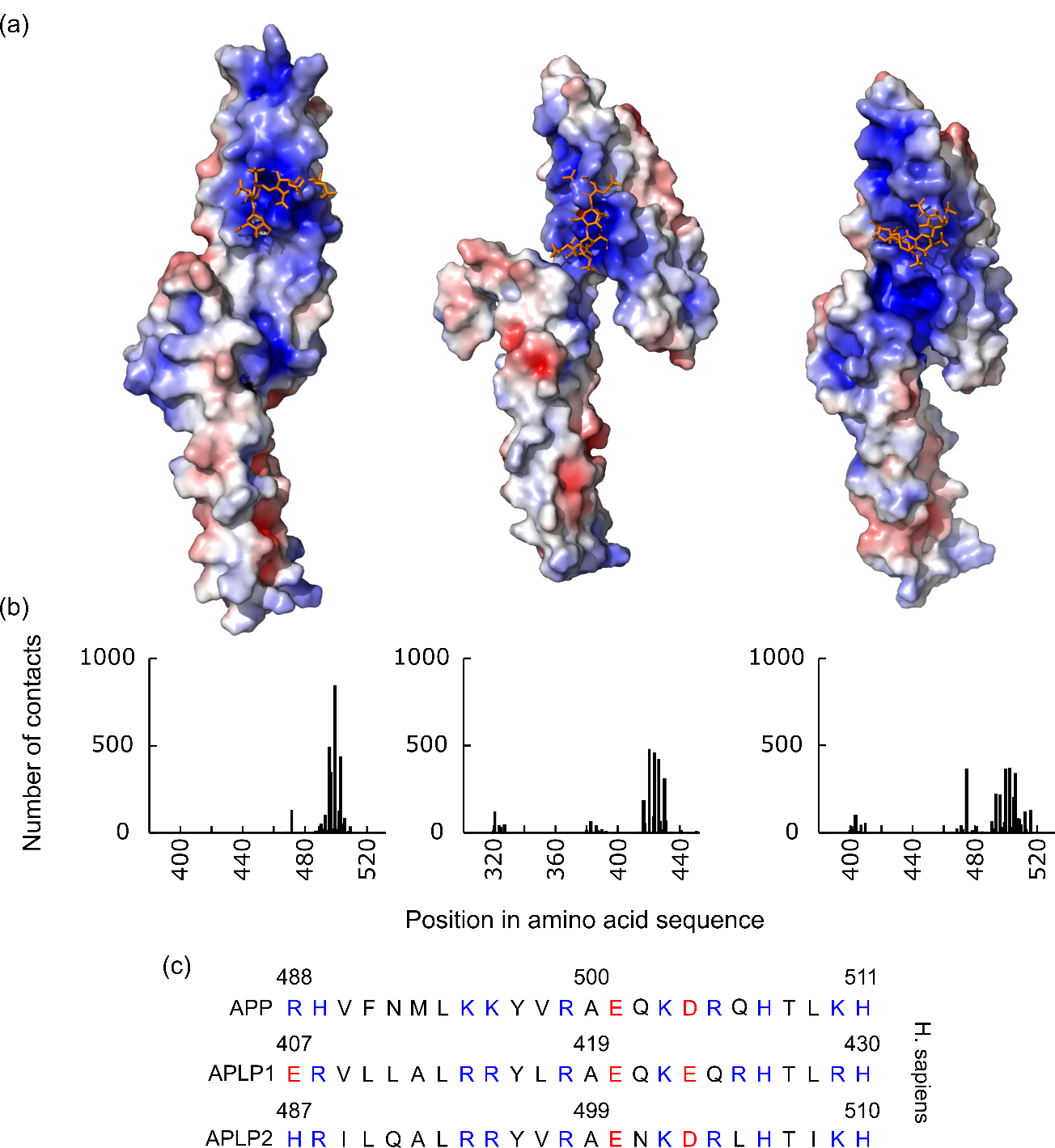

Supplement: Supplementary file 1 — Supplementary Information 1. [file 41598_2021_90737_MOESM1_ESM.docx]
